# Solitary plaque with central alopecia and scattered satellite lesions

**DOI:** 10.1016/j.jdcr.2025.06.016

**Published:** 2025-06-21

**Authors:** Reshma Gupte, Asharbh Raman

**Affiliations:** Department of Dermatology, Venereology and Leprosy, Dr. D. Y. Patil Medical College, Hospital and Research Centre, Dr. D. Y. Patil Vidyapeeth, Pune, Maharashtra, India

**Keywords:** Borderline tuberculoid leprosy, leprosy, Hansen disease, histopathology

## Case description

**Fig 1:**
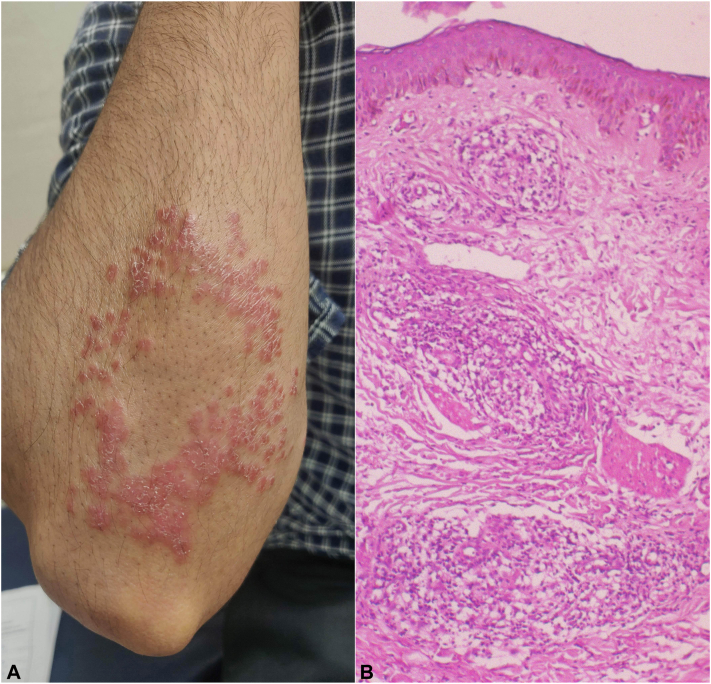
**A,** A well-defined annular erythematous plaque with fine scaling and loss of hair in the center and scattered erythematous papules around the periphery in an oval configuration over the right forearm just below elbow. **B,** Histopathology showing an atrophic epidermis with a subepidermal clear zone and granulomas in the dermis as multiple focal collections of epithelioid cells and lymphocytes. Nerve structures also show similar infiltrate (Hematoxylin and Eosin, 40×).

A 22-year-old man presented with an oval erythematous plaque characterized by irregular broken borders and scattered peripheral papules over his right forearm below the elbow (Fig. 1, *A*), which had been slowly increasing in size for the past 3 months. The lesions were associated with reduced sweating, fine scaling, noticeable loss of hair, and diminished hot and cold sensation. A thickened, nontender right ulnar nerve was also palpable. There was no evidence of sensory loss or motor function deficit over the forearm and hand. Slit skin smear did not show any acid-fast bacilli. Histopathology revealed an atrophic epidermis with a subepidermal clear zone and dermal granulomas consisting of multiple focal collections of epithelioid cells and lymphocytes. Nerve structures showed a similar infiltrate (Fig. 1, *B*). Routine blood investigations were within normal limits, and a chest radiograph revealed no abnormalities.

**Question: What is your diagnosis?**

**A.** Borderline tuberculoid leprosy
**B.** Tuberculoid leprosy
**C.** Cutaneous sarcoidosis
**D.** Lupus vulgaris
**E.** Tinea corporis

## Discussion

Correct diagnosis – Borderline tuberculoid leprosy.

Leprosy, caused by *Mycobacterium leprae*, presents with a wide variation in clinical manifestations that correlate with the host’s immunological status. Both innate and adaptive immunity play roles in its pathogenesis—the former affecting host susceptibility or resistance, and the latter having a significant effect on disease presentation.^1^ Borderline tuberculoid (BT) leprosy is part of the borderline spectrum and is associated with relatively good immunity. It is characterized by few, ill- to well-defined lesions with irregular borders, pseudopods, and satellite lesions. In contrast, solitary plaques are more common in tuberculoid (TT) leprosy, where cell-mediated immunity is strongest; the lesion is typically well-circumscribed, erythematous, and annular. Both subtypes exhibit early and prominent asymmetric nerve involvement, and associated lesions show profound sensory loss and alopecia. As the cell-mediated immunity is stronger in TT, nerve damage and resulting anesthesia are also more pronounced compared to BT.^1^^,^^2^ Histopathologically, granulomas in TT leprosy are larger and well-defined with abundant lymphocytes forming a mantle around the granuloma and multinucleated giant cells, often obscuring the subepidermal clear zone.^1^^,^^2^ In contrast, our case demonstrated granulomas that were less well-defined with fewer lymphocytes at the periphery and in clusters inside the granuloma, along with a clear subepidermal zone.

The patient in this case displayed an unusual presentation of BT leprosy with a solitary plaque, a finding usually suggestive of TT. However, the lesional morphology with pseudopods and scattered satellite lesions, as well as the findings on histopathology mentioned previously, was indispensable in reaching the correct diagnosis. A possible explanation for this presentation is that the patient initially developed TT leprosy, which then downgraded to BT leprosy due to delayed diagnosis and treatment. Various conditions may mimic leprosy, and it is important to keep the differentials in mind, especially in regions where the disease is not endemic. Unsuspecting clinicians might overlook key features, leading to misdiagnosis or delayed diagnosis and poor outcomes. Tinea corporis presenting with annular plaque and central clearing is a common misdiagnosis, especially in tuberculoid leprosy.^2^ Sarcoidosis can closely resemble leprosy both clinically and on histopathology, evident by previously reported instances of misdiagnosed BT leprosy. Molecular diagnostic techniques can be crucial in making the correct diagnosis in such cases.^3^ Cutaneous tuberculosis is another important differential to be considered, especially in endemic areas. Although a rare phenomenon, its coexistence with leprosy has also been previously reported.^4^^,^^5^

## Conflict of interest

None disclosed.
